# Altered white matter integrity in patients with monocular blindness: A diffusion tensor imaging and tract‐based spatial statistics study

**DOI:** 10.1002/brb3.1720

**Published:** 2020-06-17

**Authors:** Yu‐Xin Liu, Biao Li, Kang‐Rui Wu, Li‐Ying Tang, Qi Lin, Qing‐Hai Li, Qing Yuan, Wen‐Qing Shi, Rong‐Bin Liang, Qian‐Min Ge, Yi Shao

**Affiliations:** ^1^ Department of Ophthalmology Jiangxi Province Clinical Ophthalmology Institute The First Affiliated Hospital of Nanchang University Nanchang China; ^2^ Department of Ophthalmology Xiang’an Hospital of Xiamen University Fujian Provincial Key Laboratory of Ophthalmology and Visual Science Eye Institute of Xiamen University Xiamen University School of Medicine Xiamen China

**Keywords:** diffusion tensor imaging, monocular blindness, tract‐based spatial statistics, white matter

## Abstract

**Background:**

Visual deprivation can lead to abnormal and plastic changes in the brain's visual system and other systems. Although the secondary changes of gray matter in patients have been well studied, the study of white matter is rare. In fact, subtle changes in white matter may be revealed by diffusion tensor imaging, and tract‐based spatial statistics can be used to analyze DTI image data.

**Purpose:**

In the present study, diffusion tensor imaging (DTI) and tract‐based spatial statistics (TBSS) were used to investigate abnormal structural changes in the white matter (WM) of patients with monocular blindness (MB).

**Methods:**

We recruited 16 healthy controls (HC) (fourteen males and two females) and 16 patients (fifteen males and one female) with right‐eye blindness (without differences in left‐eye vision). All patients were of similar age. Data acquisition was performed using magnetic resonance imaging (MRI) and DTI. Voxel‐based whole brain comparisons of fractional anisotropy (FA) and radial diffusivity (RD) of WM fibers in patients and HC were performed using the TBSS method. The mean FA and RD values for altered brain regions in MB patients were analyzed via the receiver operating characteristic (ROC) curve. Correlation analysis was performed to investigate the relationships between the average FA (RD) value of the whole brain and anxiety score, depression score, and visual function questionnaire score in MB patients.

**Results:**

In MB patients, the mean FA of the whole brain was decreased versus HC. Moreover, the FA values of the corpus callosum, the corona radiata, the posterior thalamic radiation, and the right retrolenticular part of internal capsule were significantly decreased. In addition, the average RD value of the whole brain in MB patients was higher than that observed in HC. The mean FA and RD values of brain regions were analyzed using the ROC curve, and the results showed that the area under the ROC curve was more accurate. Furthermore, the average FA and RD values of the whole brain were significantly correlated with anxiety score, depression score, and visual function‐related quality of life score.

**Conclusion:**

DTI and TBSS may be useful in examining abnormal spontaneous alterations in the WM of MB patients. The observed changes in FA and RD values may imply the larvaceous neurological mechanism involved in MB.

## Graphical abstract

Diffusion tensor imaging and tract‐based spatial statistics may be useful in examining abnormal spontaneous alterations in the white matter of monocular blindness patients. The observed changes in fractional anisotropy and radial diffusivity values may imply the larvaceous neurological mechanism involved in patients with monocular blindness.

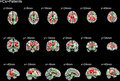

## 1 INTRODUCTION

Blindness is characterized by the loss of reaction to external light stimuli and can be classified as early or late blindness (LB). Clinically, LB is generally defined as visual deprivation occurring after the age of 12 years. There are numerous causes of blindness, including congenital and acquired. Notably, cataract is the leading cause of blindness, (Song, Wang, & Theodoratou, 2018) and glaucoma is the main cause of irreversible blindness. (Sun et al., 2019) In addition, ocular trauma and brain neuropathy may lead to blindness. (Ibrahim, Sweis, & Nockels, 2017; Koki et al., 2018) Drug and surgical treatments exert beneficial effects on reversible blindness, and recent studies have shown that treatment of irreversible blindness may be possible. (Chen et al., 2019; Shim et al., 2019) Blindness has become a global health problem, with a total of 145.2 million individuals worldwide suffering from severe vision impairment or blindness due to preventable and remediable reasons. (Di et al., 2019) This condition causes difficulties in the daily life of patients and unfavorable psychological sequelae, leading to heavy financial burden for the families and society.

Blindness is related to eye dysfunction, as well as abnormal function of the visual cortex and vision‐related cortex. Using voxel‐based morphometry, Park *et al*. (Park et al., 2009) observed that the surface range of primary and related visual areas decreased significantly in patients with congenital blindness (CB). In contrast, the surface areas of the primary visual cortex and vision‐related cortex were significantly decreased.

Diffusion tensor imaging (DTI) is an important part of magnetic resonance imaging (MRI). It can quantitatively analyze the diffusion of water molecules in tissues in three‐dimensional spaces and reveal the structure of WM and WM bundles in vivo. In recent years, MR DTI technology has been experiencing continuous progress. Measurement of abnormal changes in WM using DTI parameters has been applied to the study of LB. Fractional anisotropy (FA) and radial diffusivity (RD) are generally used to reflect the dispersion anisotropy of water molecules. Dietrich *et al*. (Dietrich, Hertrich, & Kumar, 2015) revealed that the FA of the thalamus and the right hemisphere of the thalamus were lower than those reported in healthy controls (HC), which may be suggesting that structural changes and remodeling may occur. However, several studies have shown that there is no significant difference in visual‐related brain structure and function between LB patients and HC, and brain remodeling is not clear. (Schoth, Burgel, & Dorsch, 2006; Zhang, Wan, & Ge, 2012) In addition, using DTI, Shimony *et al*. found that patients with early blindness exhibited notable atrophied visual radiation and Yu *et al*. found that integrity of the WM of corticospinal tract enhance. (Shimony et al., 2006; Yu et al., 2007) This difference is attributed to the fact that LB is not at the critical stage of visual growth at the time of visual deprivation. Therefore, the changes and remodeling of brain structure in LB are less severe compared with those observed in early blindness or CB.

Tract‐based spatial statistics (TBSS) can automatically and accurately analyze diffusion tensor data and overcome the limitations of voxel‐based morphometry in registering and smoothing. Therefore, TBSS is able to more accurately locate the abnormal areas of the WM and provide reliable parameters for the quantitative assessment of WM lesions. TBSS adopts the idea of “skeletonization,” which can align different fibers without standardization and smoothing and greatly improves the accuracy of comparisons between groups. (Abe et al., 2010; Smith et al., 2006) Wang *et al. (*Wang et al., 2013) directly compared changes in the FA values of WM in patients with CB and LB. They found that the mechanism of FA changes in the WM of CB and LB patients was different, and the loss of WM integrity was a significant feature of blindness. These studies showed that binocular blindness may lead to changes in brain anatomy and function. However, the role of monocular blindness (MB) in the introduction of abnormal changes in the brain has not been well studied. Li *et al*. and Huang *et al*. used the amplitude of low‐frequency fluctuation, voxel‐wise degree centrality, and regional homogeneity methods, respectively, to investigate abnormal brain functional changes in MB. They concluded that abnormal spontaneous changes may occur in multiple visual and vision‐related brain regions of MB patients. (Huang et al., 2017; Li et al., 2016) Using the voxel‐mirrored homotopic connectivity method, Shao *et al*. (Shao et al., 2018) found that MB patients showed abnormal functional connectivity between the cerebral hemispheres in the visual pathway. In addition, the modes of brain interhemispheric functional connectivity differed between left‐ and right‐eye MB.

The purpose of the present study was to investigate the spontaneous changes of brain structure in late‐stage MB patients versus HC using DTI and TBSS methods, which provides a new direction for the study of alteration in the WM of MB patients and may reveal the potential neurological mechanisms involved in MB.

## 2 MATERIAL AND METHODS

### 2.1 Participants

Sixteen patients (fifteen males and one female) with right‐eye MB (without significant difference in the left eye) were recruited from the First Affiliated Hospital of Nanchang University Hospital (Nanchang, China). The following criteria were used for the diagnosis of MB: (a) eyeball burst caused by trauma and received eyeball removal within 48 hr after the trauma; (b) late stage of MB; and (c) normal contralateral eye without any other ophthalmic disease (cataracts, glaucoma, optic neuritis, and retinal degeneration).

The exclusion criteria were as follows: (a) CB; (b) contralateral visual impairment; (c) blindness caused by eye diseases (e.g., cataracts, glaucoma, optic neuritis, macular degeneration, and ocular ischemic diseases); (d) history of binocular surgery; (e) medical treatment for blindness; (f) cerebral infarction (e.g., cerebral hemorrhage and cerebral infarction); and (g) presence of mental disorders.

In addition, the study included 16 HC participants (14 males and 2 females), matched with those of the MB group in terms of age and educational level. The inclusion criteria for the HC were as follows: (a) absence of neurological abnormalities or psychiatric disorders; (b) with 20/20 vision; (c) no ophthalmic diseases; and (d) no contraindications to MRI scanning. The subjects average age was 50.12, right‐handed, and had a high school education or above. The normal naked eye vision of the patients was consistent with that of the control group, and their eye dominance was matched.

The study was approved by the Medical Ethics Committee of the First Affiliated Hospital of Nanchang University. Prior to providing informed consent, all participants understood the purpose, methods, and potential risks.

### 2.2 Data acquisition

All MRI data were taken from the Department of Radiology of the First Affiliated Hospital of Nanchang University in China with a 3.0‐T MRI scanner (Siemens, Erlangen, Germany) equipped with an 8‐channel phased‐array head coil. The data acquisition in this study included a three‐dimensional T1‐weighted magnetization prepared rapid acquisition gradient echo (MPRAGE) scan and a DTI spin echo plane scan. The following were parameters for those sequences:

T1‐weighted images: repetition time (TR) = 1900 ms, echo time (TE) = 2.26 ms, flip angle = 9°, field of view (FOV) = 240 × 240 mm, matrix = 256×256, slice thickness = 1 mm, and sagittal slice number = 176.

DTI spin echo planar images: stereo pixel size = 1×1 × 1 mm^3^, TR = 8,000 ms, TE = 89 ms, FOV = 250×250 mm, matrix slice thickness = 2 mm, slice numbers = 62, direction = axial, 64 nonlinear diffusion weighting gradient directions with b = 1,000 s/mm^2^, and an additional b0 (b value = 0) image.

### 2.3 Data preprocessing

The FMRIB Software Library (FSL) (http:// www.fmrib.ox.ac.uk/fsl) was used to preprocess the MRI data. First, all the original data of the brain were extracted and collected. Second, we utilized the v3.0 FMRIB Diffusion Toolbox (FDT) (http://fsl.fmrib.ox.ac.uk/fsl/fslwiki/FDT) to reduce the impact of head motion artifacts. Subsequently, we utilized the script Brain Extraction Tool based on the b0 image to create a brain mask. Finally, we utilized the v3.0 FTD software (http://fsl. fmrib.ox.ac.uk/fsl/fslwiki/FTD) in the FSL to reconstruct the tensor matrix of the original diffusion data and calculated the diffusion scalar measurements, including FA and RD.

### 2.4 TBSS procedures

The following data processing techniques were used to analyze the white matter diffusion characteristics, as described in the previous TBSS method report. In spite of the fact that the white matter skeleton was generated according to FA pictures, TBSS analysis was performed, respectively, on FA and RD. All FA pictures were aligned to a Montreal Neurological Institute 152 (MNI152) standard space (1 × 1 × 1 mm^3^) through nonlinear registration, in which the FMRIB58_FA standard‐space image (http://fsl.fmrib.ox.ac.uk/fsl/fslwiki/FMRIB58_FA) was considered as the template.

To build an average FA skeleton, the average FA pictures of all participants were projected onto the FMRIB58_skeleton. The FA threshold value was set at 0.2 to include principal fascia and eliminate surrounding tracts in the case of variations between subjects or partial volume effect interference. In order to realize nonlinear registration and skeletonization, RD were separately projected onto the original average FA skeleton obtained from the FA registration and skeletonization. After aligning with the common skeleton, the data appear as a four‐dimensional image. The Johns Hopkins University (JHU) white matter tractography atlas and the JHU International Consortium of Brain Mapping Li et al. 1965 (ICBM)‐DTI‐81 white‐matter labels were used to identify specific fiber bundles.

Subsequently, the FSL clustering tool was used to extract the statistically significant voxel clusters of FA and RD. (Chen, Gao, Che, Lin, & Ruan, 2018) The script tbss_fill was used to fill in blocks, making it easier to visualize the manifestation of realistic analysis.

### 2.5 Assessment of anxiety, depression, and visual quality of life

The scores of anxiety and depression were investigated by the Chinese version of 1983 HADS (The Hospital Anxiety and Depression Scale) designed by Zigmond AS and Snaith RP in 1983, which was conductive to detecting and managing emotional disorder in patients in medical and surgical departments. (Carlson, Silva, & Conboy, 2008) The quality of life score of MB patients was measured by the Chinese version of NEI‐VFQ25 (25‐item National Eye Institute Visual Function Questionnaire) in terms of general health and vision acuity, the grade of activity disability, and the reflection of vision problem. (Mangione et al., 1998, 2001) Questionnaire survey was conducted on the subjects, and the subjects were asked to respond to their psychological status in the last month (one month before surgery).

### 2.6 Receiver operating characteristic curve

We speculated that abnormal changes in FA and RD values might be important diagnostic symbols for MB. ROC curves were used to analyze the mean FA and RD values of altered brain regions in MB patients. The area under the ROC curve (AUC) represents the diagnosis rate. AUC was positively proportional to the diagnostic rate. AUC ranges from 1.0 to 0.5. And the AUC value of 0.7 to 0.9 expresses a confident accuracy and higher than 0.9 indicates high accuracy.

### 2.7 Statistical analysis

To compare the demographic and clinical parameters of the two groups, IBM SPSS statistical software, version 23.0 (IBM Corp., Armonk, NY, USA), was used with two‐independent‐sample *t* tests (*p* < .05). In our statistical analysis, the permutation‐based nonparametric method is implemented by Threshold‐Free Clustering Enhancement option in the FSL randomization tool. The random permutation was set at 5,000; moreover, age was regressed as covariates. A grade of *p* < .05 suggested that there was statistically significance, which was completely corrected after several comparisons.

## 3 RESULTS

### 3.1 TBSS differences

**Figure 1:**
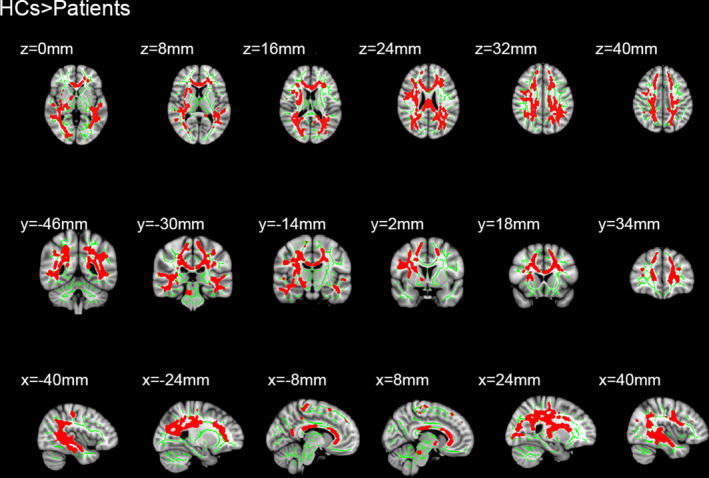
Results of whole‐brain tract‐based spatial statistics analysis comparing fractional anisotropy between patients with monocular blindness and healthy controls. Significantly decreased fractional anisotropy values of MB group were shown in the corpus callosum, the right retrolenticular part of internal capsule, the anterior corona radiate, the superior corona radiate, the posterior corona radiate, the posterior thalamic radiation, the right sagittal stratum (right), and the superior longitudinal fasciculus. The skeleton image (green = RD > 0.2) was overlaid by the mean fractional anisotropy image. HC, healthy controls; MB, monocular blindness

**Figure 2:**
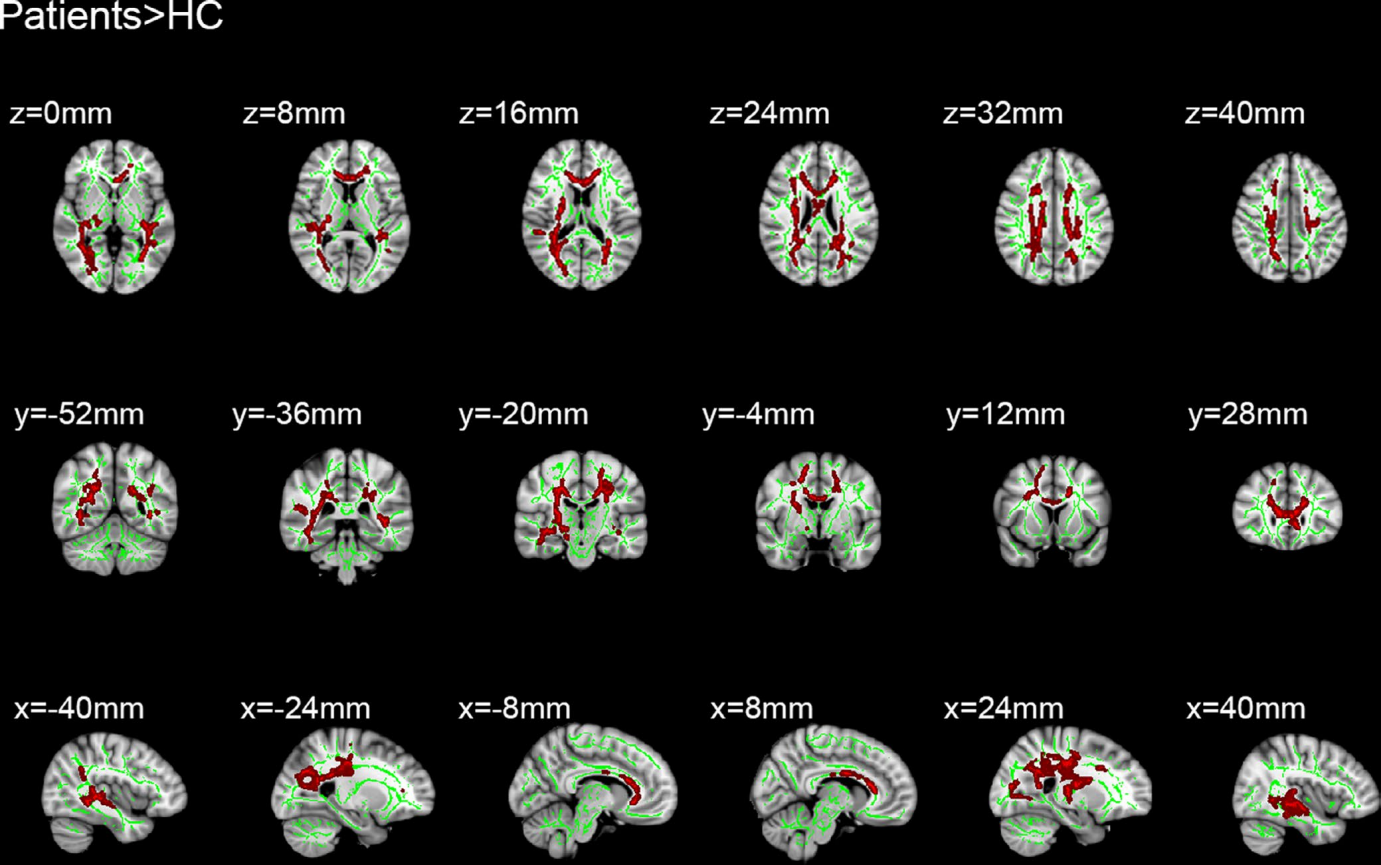
Comparison of radial diffusivity in patients with monocular blindness and healthy controls. The statistically significant clusters are presented at different coordinates in these parts: the corpus callosum, the left posterior thalamic radiation, the right superior corona radiate, the right posterior corona radiate, the sagittal stratum, and the left superior longitudinal fasciculus. And the red areas indicate all tracts with significantly increased RD values in the MB group, which may reflect abnormal white matter integrity (*p* < .05). HCs, healthy controls; MB, monocular blindness; RD, radial diffusivity

Sixteen patients with MB and 16 HC were included in this study. Of note, there were no distinct differences in terms of age between the two groups. Significantly different voxel clusters between the MB and HC groups were found in FA and RD values. In MB patients, the mean FA of the whole brain decreased versus the HC. Furthermore, the FA values of the corpus callosum (CC), corona radiata (CR), posterior thalamic radiation (PTR), superior longitudinal fasciculus (SLF), the right retrolenticular part of internal capsule, and the right sagittal stratum (SS) were significantly decreased (Table 1 and Figure 1). In addition, the average RD value of the whole brain in MB patients was higher than that observed in HC. The RD values were significantly increased in the body and genu of CC, SS, left anterior corona radiata (ACR), left PTR, left SLF, superior corona radiata (SCR), and posterior corona radiata (PCR) in the right brain (Table 2 and Figure 2).

**Table 1:** Conditions of participants included in the study

Condition	MB	HCs	*t*	*p*‐value*
Male/female	15/1	14/2	0.169	.982
Age (years)	50.12 ± 5.34	51.26 ± 5.76	0.282	.882
Weight (kg)	65.12 ± 9.81	63.89 ± 10.09	0.193	.939
Handedness	16R	16R	*N*/A	>.99
Duration of DON (days)	51.24 ± 6.64	*N*/A	*N*/A	*N*/A
Best‐corrected Va‐left eye	0.95 ± 0.20	1.05 ± 0.25	−0.158	.928
Best‐corrected Va‐right eye	–	1.05 ± 015	*N*/A	*N*/A

Abbreviations: HCs, healthy controls; MB: monocular blindness; *N*/A, not applicable.

* *p* < .05 Independent *t* tests comparing two groups.

**Table 2:** Clusters showing significant differences in RD between patients and HCs

Variable	Comparison	TFCE corrected *p*	Cluster number/size	MNI atlas coordinates			Tract(s) with in clusters
				*X*	*Y*	*Z*	
RD	HCs < Patients	<0.01	1/158	73	108	107	Corpus callosum
			2/224	116	66	86	Posterior thalamic radiation(left) Superior corona radiate (right) Posterior corona radiata (right)
			3/121	128	79	66	Sagittal stratum
			4/98	128	76	93	Superior longitudinal fasciculus (left)

Note

The statistical threshold was set at voxel level with *p* < .05 for multiple comparisons using Gaussian random field theory voxels with *p* < .01 and cluster size > 40 voxels, AlphaSim corrected.

Abbreviations: FA, fractional anisotropy; HCs, healthy controls; MNI, Montreal Neurological Institute; RD, radial diffusivity; TFCE corrected *p*, Threshold‐Free Cluster Enhancement corrected *p* value.

### 3.2 Receiver operating characteristic curve

**Figure 3:**
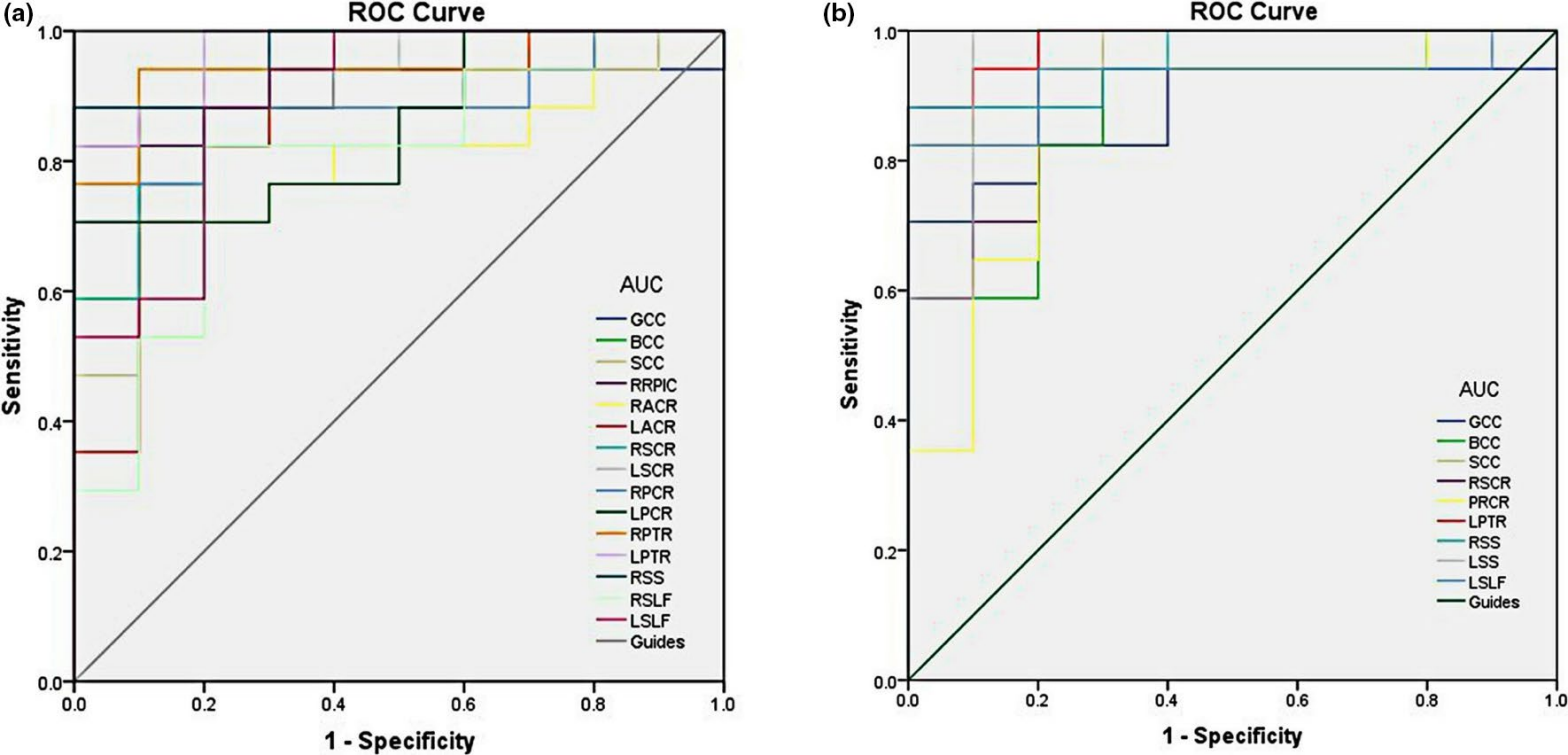
ROC curve analysis of the mean FA and RD values for altered brain regions. (a) The area under the ROC curve of the FA values was 0.876 (*p* < .001; 95% CI: 0.741–1.000) for GCC, BCC 0.871 (*p <* .001; 95% CI: 0.733–1.000), SCC 0.959 (*p* < .001; 95% CI: 0.893–1.000), RSCR 0.929 (*p* < .001; 95% CI: 0.827–1.000), RPCR 0.856 (*p* > .05; 95% CI: 0.712–1.000), LPTR 0.982 (*p < *.001; 95% CI: 0.944–1.000). RSS, 0.959 (*p *< .001; 95% CI: 0.891–1.000), LSS, 0.959 (*p *< .001; 95% CI: 0.876–1.000), LSLF, 0.924 (*p *< .001; 95% CI: 0.814–1.000). (b) The area under the ROC curve of the RD values was 0.894 (*p < *.001; 95% CI: 0.766–1.000) for GCC, BCC 0.882 (*p *< .001; 95% CI: 0.755–1.000), SCC 0.935 (*p* < .001; 95% CI: 0.846–1.000), RRPIC 0.924 (*p *< .001; 95% CI: 0.823–1.000), RACR 0.794 (*p *< .001; 95% CI: 0.624–0.964), LACR 0.853 (*p *< .001; 95% CI: 0.697–1.000), RSCR 0.935 (*p *< .001; 95% CI: 0.841–1.000), LSCR 0.876 (*p *< .001; 95% CI: 0.740–1.000), RPCR 0.888 (*p *< .001; 95% CI: 0.761–1.000), LPCR 0.853 (*p *< .001; 95% CI: 0.711–0.995), RPTR 0.941 (*p *< .001; 95% CI: 0.850–1.000), LPTR 0.971 (*p *< .001; 95% CI: 0.917–1.000), RSS 0.965 (*p *< .001; 95% CI: 0.904–1.000), RSLF 0.794 (*p *< .001; 95% CI: 0.616–0.973), LSLF 0.894 (*p *< .001; 95% CI: 0.767–1.000). AUC, area under the curve; FA, fractional anisotropy; RD, radial diffusivity; ROC, receiver operating characteristic; GCC, genu of corpus callosum; BCC, body of corpus callosum; SCC, splenium of corpus callosum; RSCR, right superior corona radiata; RPCR, right posterior corona radiata; LPTR, left posterior thalamic radiation; RSS, right sagittal stratum; LSS, left sagittal stratum; LSLF, left superior longitudinal fasciculus; RRPIC, right retrolenticular part of internal capsule; RACR, right anterior corona radiata; LACR, left anterior corona radiata; LSCR, left superior corona radiata; LPCR, left posterior corona radiata; RPTR, right posterior thalamic radiation; RSLF, right superior longitudinal fasciculus

The areas under the curve for the FA values were as follows: genu of CC, 0.876 (*p* < .001; 95% confidence interval (CI): 0.741–1.000); body of CC, 0.871 (*p* < .001; 95% CI: 0.733–1.000); splenium of CC, 0.959 (*p* < .001; 95% CI: 0.893–1.000); right SCR, 0.929 (*p* < .001; 95% CI: 0.827–1.000); right PCR, 0.856 (*p* > .05; 95% CI: 0.712–1.000); left PTR, 0.982 (*p* < .001; 95% CI: 0.944–1.000); right SS, 0.959 (*p* < .001; 95% CI: 0.891–1.000); left SS, 0.959 (*p* < .001; 95% CI: 0.876–1.000); and left SLF, 0.924 (*p* < .001; 95% CI: 0.814–1.000) (Figure 3).

The areas under the curve for the RD values were as follows: genu of CC, 0.894 (*p* < .001; 95% CI: 0.766–1.000); body of CC, 0.882 (*p* < .001; 95% CI: 0.755–1.000); splenium of CC, 0.935 (*p* < .001; 95% CI: 0.846–1.000); right retrolenticular part of internal capsule, 0.924 (*p* < .001; 95% CI: 0.823–1.000); right ACR, 0.794 (*p* < .001; 95% CI: 0.624–0.964); left ACR, 0.853 (*p* < .001; 95% CI: 0.697–1.000); right SCR, 0.935 (*p* < .001; 95% CI: 0.841–1.000); left SCR, 0.876 (*p* < .001; 95% CI: 0.740–1.000); right PCR, 0.888 (*p* < .001; 95% CI: 0.761–1.000); left PCR, 0.853 (*p* < .001; 95% CI: 0.711–0.995); right PTR, 0.941 (*p* < .001; 95% CI: 0.850–1.000); left PTR, 0.971 (*p* < .001; 95% CI: 0.917–1.000); right SS, 0.965 (*p* < .001; 95% CI: 0.904–1.000); right SLF, 0.794 (*p* < .001; 95% CI: 0.616–0.973); and left SLF, 0.894 (*p* < .001; 95% CI: 0.767–1.000) (Figure 3 and Table 3).

**Table 3:** Clusters showing significant differences in FA between patients and HCs

Variable	Comparison	TFCE corrected *p*	Cluster Number/size	MNI atlas coordinates			Tract(s) with in clusters
				*X*	*Y*	*Z*	
FA	HCs > Patients	<0.01	1/292	117	101	63	Corpus callosum Retrolenticular part of internal capsule (right) Anterior corona radiata Superior corona radiata Posterior corona radiata Posterior thalamic radiation Sagittal stratum (right) Superior longitudinal fasciculus

Note

The statistical threshold was set at voxel level with *p* < .05 for multiple comparisons using Gaussian random field theory voxels with *p* < .01 and cluster size > 40 voxels, AlphaSim corrected.

Abbreviations: FA, fractional anisotropy; HCs, healthy controls; MNI, Montreal Neurological Institute; TFCE corrected *p*, Threshold‐Free Cluster Enhancement corrected *p* value.

### 3.3 Correlation analysis

**Figure 4:**
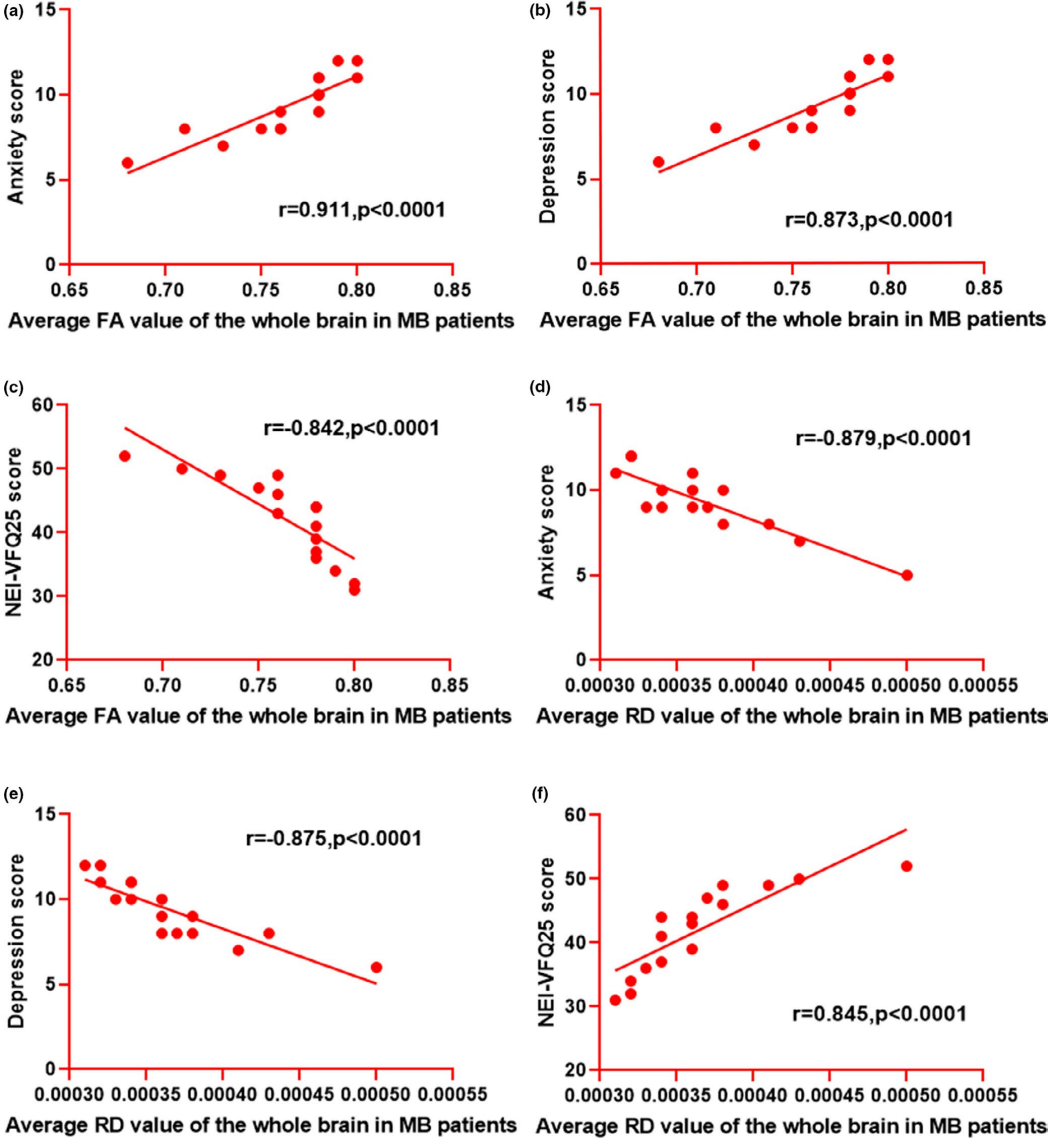
Correlation between the average FA (RD) values of the whole brain in MB patients and anxiety, depression, and NEI‐VFQ25 scores. (a) The FA value was positively correlated with anxiety score (*r* = .911, *p* < .0001). (b) The FA value was positively correlated with depression score (*r* = .873, *p* < .0001). (c) The FA value was negatively correlated with NEI‐VQF25 score (*r* = −.842, *p* < .0001). (d) The RD value showed a negative correlation with anxiety score (*r* = −.879, *p* < .0001). (e) The average RD value of the whole brain showed a negative correlation with depression score (*r* = −.875, *p* < .0001). (f) The RD value showed a positive correlation with NEI‐VQF25 score (*r* = −.842, *p* < .0001). FA, fractional anisotropy; RD, radial diffusivity; NEI‐VFQ25, 25‐item National

In the MB group, the average FA value of the whole brain was positively correlated with the anxiety score (*r* = .911, *p* < .0001) and depression score (*r* = .873, *p* < .0001). However, it was inversely associated with the NEI‐VQF25 score (*r *= −.842, *p* < .0001). The average RD value of the whole brain displayed negative correlations with the anxiety score (*r *= −.879, *p* < .0001) and depression score (*r *= −.875, *p* < .0001). However, it showed a positive correlation with the NEI‐VQF25 score (*r *= −.842, *p* < .0001) (Figure 4).

## 4 DISCUSSION

In the present study, DTI and TBSS were used to analyze and compare the structure of WM fiber bundles in MB patients and HC. We found that, among significantly different voxel clusters, MB patients exhibited reduced FA values and increased RD values versus HC.

We used voxel‐wise statistics with threshold‐free cluster enhancement to obtain the FA and RD values. FA values are quantitative indicators of the structural integrity of WM fibers. In addition, an increase in RD values may reflect an increase in neuronal branches. (Silk, Vance, & Rinehart, 2009).

The CC is the major lacertus in the brain, mainly connecting homologous cortical areas in the two hemispheres. Pietrasanta *et al*. (Pietrasanta, Restani, & Caleo, 2012) showed that deprivation of sensory experience may alter the fine structure of the CC, as well as regulate the function and remodeling of the visual cortex. Moreover, they found that the role of interhemispheric connections in visual perception was complex. In addition, studies have shown that abnormal changes in the CC are related to various congenital oculopathies, such as primary congenital glaucoma and posterior polymorphous corneal dystrophy. (Gunawan, Komaratih, & Nurwasis, 2018; Jang, Roldan, & Frausto, 2014) Therefore, we can infer that changes in the CC of MB patients are closely related to visual deprivation. Malavolti *et al*. (Malavolti et al., 2017) found that the severity of WM injury was closely associated with the development of the CC. Moen *et al*. (Moen, Håberg, & Skandsen, 2014) found that the CC exhibited structural changes after moderate‐to‐severe brain injury, and they thought that these effects may be related to the ability to perform rapid and complex sensorimotor movements. Perhaps the impairment of movement due to visual deprivation causes tiny degenerative changes in the CC. Barone *et al*. (Barone et al., 2018) indicated that the integrity of the CC structure was closely associated with cognitive ability. However, there is evidence that blind individuals use cognitive mechanisms differently versus healthy individuals. (Cattaneo et al., 2008) This suggests that there is a mechanism of compensation for the cognitive limitations linked to visual impairment. In this study, the decreased FA and increased RD values of the CC may explain the structural and functional remodeling of the brain after visual deprivation.

The visual cortex in each hemisphere is connected with the other hemisphere via axonal projections passing through the splenium of the CC. (Saenz & Fine, 2010) Shi *et al*. (Shi et al., 2015) affirmed that there was an obvious difference around the splenium region between the sighted and blind groups. Moreover, there was a particular difference between LB patients and CB patients. Using diffusion‐weighted MRI, previous studies showed that the splenium of the CC in patients suffering from various visual deformations exhibits white matter damage. (Nagaishi et al., 2015; Singhi, Saini, & Sankhyan, 2015; Winslow, Mickey, & Frohman, 2006) Therefore, changes in the FA and RD values of the splenium of the CC in MB patients are important to study the mechanism of visual deprivation and brain remodeling.

Corona radiate refers to the radial fibrous between the internal capsule and the cerebral cortex. The anterior corona radiata connects cerebral cortex with the brain stem. Gaudio *et al*. (Gaudio et al., 2017) demonstrated that the ACR was involved in cognitive control, as well as visual perception and processing. Maruta *et al*. (Maruta, Suh, & Niogi, 2010) found that gaze errors affect attention and working memory, which are related to variation in the structure of the ACR bundle. A previous study showed that differences in connectivity between the left ACR and the right lenticular nucleus may mediate the effects of genotypes on audiovisual speech perception and reveal the pathogenesis of autism. (Ross et al., 2017) Therefore, we infer that changes in the structure of the ACR bundle are closely related to the loss of visual function in MB patients. Li *et al*. (Li et al., 2014) used DTI to detect secondary lesions of WM at the left frontal of patients with acute left coronary artery infarction. Therefore, secondary changes in the ACR may be related to changes in the executive ability of MB patients. Karababa *et al*. (Karababa et al., 2015) indicated that abnormal emotional regulation may be attributed to abnormal WM changes in fiber bundle length and fiber density in the ACR. Changes in the FA and RD values suggest that MB patients may exhibit affective disorders. Sanjuan *et al*. (Sanjuan, Thoma, & Claus, 2013) found that the FA value of the ACR was correlated with the severity of post‐traumatic stress. Therefore, part of the brain remodeling may be induced in MB patients through stress.

Research has shown that the integrity of the thalamus connection is gradually damaged in parallel with a decline in cognitive function. (Zhu et al., 2015) It was found that the decreased volume of bilateral lateral geniculate bodies and visual radiation in CB may imply the formation of new heterotopic junctions between the thalamic sensory nucleus and lateral geniculate bodies. Notably, this conclusion has been preliminarily confirmed in animal models. (Cecchetti et al., 2016; Karlen, Kahn, & Krubitzer, 2006) Rose *et al. (*Rose et al., 2014) found that the FA values of the neonatal internal capsule increased, and the internal capsule was the early formation area of the neonatal myelin sheath. The decrease of the FA value in the internal capsule of MB patients indicates that the structure of the myelin sheath is damaged after visual deprivation. Therefore, this decrease may reflect the mechanism involved in brain structural damage and functional changes.

A previous study has shown that the decreased number of branches of the thalamocortical afferents to the somatosensory cortex may lead to disorders of sensory information processing. (Lee, 2009) Increased RD values reflect an increase in neuronal branches, so the increase of the RD values in the thalamus may be related to the compensatory ability of MB patients for sensory information processing. Our hypothesis is that other regions of the brain may also attempt to compensate for damage of the nerve tract by increasing the number of neural branches, thereby improving the functions mediated by these cortices.

Previous studies have shown that patients with diabetic retinopathy, glaucoma, and strabismus exhibit psychological problems and a reduced quality of life associated with vision. (Alpak et al., 2014; Wu, Kong, & Gao, 2019; Yu et al., 2013) Therefore, we studied the association of the mean FA and RD values of the whole brain in MB patients with the anxiety and depression scores measured by the Hospital Anxiety and Depression Scale, and we also studied the association of the values with quality of life related to visual function measured using the NEI‐VQE25. We found that higher FA values and lower RD values were linked to the development of a serious psychological disorder and worsening of the quality of life of MB patients, respectively. Coloigner *et al. (*Coloigner et al., 2019) reported that depression was closely related to FA and RD values in the splenium of the CC and posterior limbs of the internal capsule. In addition, increased anxiety was associated with FA values in the genu and splenium of the CC, ACR, and PTR. Using 3.0 Tesla MRI scans, as well as neuropsychological and quality of life assessment in patients with confluent WM hyperintensities, Croall *et al. (*Croall et al., 2017) confirmed the correlation between DTI parameters and cognition. Both visual impairment and visual field defects increase the risk of psychological disorders in MB patients and significantly reduce their quality of life.

This study was characterized by limitations. First, the small sample size may affect the reliability of the results. (Button et al., 2013) Second, there are numerous clinical causes of blindness, and it has been shown that FA and RD are related to heredity. (Hatton et al., 2018) Therefore, this study may not be able to accurately evaluate the results of the TBSS measurements in MB patients, owing to individual differences. Further research is warranted to explain the relationship between the course of disease and changes in the WM and investigate the interaction of the FA and RD values.

TBSS is more accurate in identifying WM abnormalities. Therefore, this method has been applied in the clinical study of comitant exotropia, schizophrenia, multiple sclerosis, and other diseases. However, TBSS continues to be characterized several limitations. (Li et al., 2018; Mamah, Ji, & Rutlin, 2019) First, TBSS is only suitable for the detection of large WM lacertus. (Focke et al., 2008) Second, the FA skeleton at the intersection of fibers tends to break. (Smith et al., 2006; Tuch, Salat, & Wisco, 2005) Moreover, TBSS may not eliminate the influence of head movement during scanning. (Smith et al., 2006) Additional studies should address these shortcomings. Shortly, using TBSS to analyze the abnormal spontaneous changes of WM in patients provides a better method for us to study the larvaceous neurological mechanism of MB.

## 5 CONCLUSION

In a conclusion, we found that DTI and TBSS may be useful in examining abnormal spontaneous alterations in the WM of MB patients. And the observed changes in FA and RD values may imply the larvaceous neurological mechanism involved in MB.

## CONFLICT OF INTEREST

This was not an industry supported study. All the authors report no conflict of interests in this work.

## AUTHORS' CONTRIBUTIONS

Yi Shao, Kang‐Rui Wu, and Biao Li designed and conducted of the current study; Li‐Ying Tang, Qi Lin, Qing‐Hai Li, Qing Yuan, Wen‐Qing Shi, Rong‐Bin Liang, and Qian‐Min Ge collected, managed, analyzed, and interpreted the data; and Yu‐Xin Liu wrote the manuscript. All the authors read and approved the final manuscript, and Yi Shao made a decision to submit the manuscript for publication.

## DATA AVAILABILITY STATEMENT

The data that support the findings of this study are available from the corresponding author upon reasonable request.
